# The Clinical Usefulness of Predictive Models for Preterm Birth with Potential Benefits: A KOrean Preterm collaboratE Network (KOPEN) Registry-Linked Data-Based Cohort Study

**DOI:** 10.7150/ijms.37626

**Published:** 2020-01-01

**Authors:** Kyung Ju Lee, Jinho Yoo, Young-Han Kim, Soo Hyun Kim, Seung Chul Kim, Yoon Ha Kim, Dong Wook Kwak, Kicheol Kil, Mi Hye Park, Hyesook Park, Jae-Yoon Shim, Ga Hyun Son, Kyung A Lee, Soo-young Oh, Kyung Joon Oh, Geum Joon Cho, So-yeon Shim, Su Jin Cho, Hee Young Cho, Hyun-Hwa Cha, Sae Kyung Choi, Jong Yun Hwang, Han-Sung Hwang, Eun Jin Kwon, Young Ju Kim, the KOrean Preterm collaboratE Network (KOPEN) Working Group

**Affiliations:** 1 Department of Obstetrics and Gynecology, Korea University Medical Center, Seoul, Korea; 2 Department of Public Health, Korea University Graduate School, Seoul, Korea; 3 YooJin BioSoft Co., Ltd, Goyang-si Gyeonggi-do, Korea; 4 Department of Obstetrics and Gynecology, Institute of Women's Life Medical Science, Yonsei University College of Medicine, Seoul, Korea; 5 Department of Obstetrics & Gynecology, CHA Gangnam Medical Center, CHA University, Seoul, Korea; 6 Department of Obstetrics and Gynecology, Biomedical Research Institute, Pusan National University College of Medicine, Busan, Korea; 7 Department of Obstetrics and Gynecology, Chonnam National University Medical School, Gwangju, Korea; 8 Department of Obstetrics and Gynecology, Cheil General Hospital and Woman's Healthcare Center, Dankook University College of Medicine, Seoul, Korea; 9 Department of Obstetrics and Gynecology, Ajou University School of Medicine, Suwon, Korea; 10 Department of Obstetrics and Gynecology, College of Medicine, Catholic University of Korea, Seoul, Korea; 11 Department of Obstetrics and Gynecology, College of Medicine, Ewha Womans University, Seoul, Korea; 12 Department of Preventive Medicine, College of Medicine, Ewha Womans University, Seoul, Korea; 13 Department of Obstetrics & Gynecology, Asan Medical Center, University of Ulsan College of Medicine, Seoul, Korea; 14 Department of Obstetrics and Gynecology, Kangnam Sacred Heart Hospital, Hallym University College of Medicine, Seoul, Korea; 15 Department of Obstetrics and Gynecology, Kyung Hee University School of Medicine, Seoul, Korea; 16 Department of Obstetrics and Gynecology, Samsung Medical Center, Sungkyunkwan University School of Medicine, Seoul, Korea; 17 Department of Obstetrics and Gynecology, Seoul National University Bundang Hospital, Seongnam, Korea; 18 Department of Obstetrics and Gynecology, Korea University Medical Center, Seoul, Korea; 19 Department of Pediatrics, College of Medicine, Ewha Womans University, Seoul, Korea; 20 Department of Obstetrics and Gynecology, CHA Bundang Medical Center, CHA University School of Medicine, Seongnam, Korea; 21 Department of Obstetrics & Gynecology, Kyungpook National University Hospital, Kyungpook National University, School of Medicine, Daegu, Korea; 22 Department of Obstetrics and Gynecology, College of Medicine, Catholic University of Korea, Seoul, Korea; 23 Department of Obstetrics and Gynecology, Kangwon National University School of Medicine, Kangwon-do, Korea; 24 Department of Obstetrics and Gynecology, Research Institute of Medical Science, Konkuk University School of Medicine, Seoul, Korea.

**Keywords:** Preterm birth, Prediction model, Risk factor

## Abstract

**Background**: Preterm birth is strongly associated with increasing mortality, incidence of disability, intensity of neonatal care required, and consequent costs. We examined the clinical utility of the potential preterm birth risk factors from admitted pregnant women with symptomatic preterm labor and developed prediction models to obtain information for prolonging pregnancies.

**Methods:** This retrospective study included pregnant women registered with the KOrean Preterm collaboratE Network (KOPEN) who had symptomatic preterm labor, between 16 and 34 gestational weeks, in a tertiary care center from March to November 2016. Demographics, obstetric and medical histories, and basic laboratory test results obtained at admission were evaluated. The preterm birth probability was assessed using a nomogram and decision tree according to birth gestational age: early preterm, before 32 weeks; late preterm, between 32 and 37 weeks; and term, after 37 weeks.

**Results:** Of 879 registered pregnant women, 727 who gave birth at a designated institute were analyzed. The rates of early preterm, late preterm, and term births were 18.16%, 44.02%, and 37.83%, respectively. With the developed nomogram, the concordance index for early and late preterm births was 0.824 (95% CI: 0.785-0.864) and 0.717 (95% CI: 0.675-0.759) respectively. Preterm birth was significantly more likely among women with multiple pregnancy and had water leakage due to premature rupture of membrane. The prediction rate for preterm birth based on decision tree analysis was 86.9% for early preterm and 73.9% for late preterm; the most important nodes are watery leakage for early preterm birth and multiple pregnancy for late preterm birth.

**Conclusion:** This study aims to develop an individual overall probability of preterm birth based on specific risk factors at critical gestational times of preterm birth using a range of clinical variables recorded at the initial hospital admission. Therefore, these models may be useful for clinicians and patients in clinical decision-making and for hospitalization or lifestyle coaching in an outpatient setting.

## Introduction

The overall spontaneous and iatrogenic preterm birth rates showed clinically varied country-specific rates between 5% to 13% per year over the past few decades 1-3. In Korea, preterm birth rates have increased over 1.5 times between 2007 and 2017 4. The World Health Organization (WHO) categorizes preterm births based on the gestational age as follows: extremely preterm (<28 weeks), very preterm (28-32 weeks), and moderate or late preterm (32-37 weeks) 5, 6. An earlier preterm birth is strongly associated with increasing mortality, incidence of disability, intensity of neonatal care required, and consequent costs 1, 7.

Identifying women at risk of preterm birth is an important task for clinical care providers. However, there are only a few methods available for reliably predicting actual preterm labor in women who present with symptoms of labor. Currently, pregnant women with symptomatic preterm labor undergo a transvaginal ultrasound examination and cervicovaginal fetal fibronectin test, but these yield a very high false-positive rate which leads to increased unnecessary hospitalizations and administration of tocolytics and glucocorticoids 8, 9.

As a part of the preterm birth management entry process, electronic systems, such as the clinical decision support systems, help determine the risk for a range of medical conditions, directly affecting the decision making and the individual patient-specific assessment and counseling. The use of these systems is effective and has a significant impact on the improvement of clinical practice 10, 11. In 2017, the American College of Obstetricians and Gynecologists recommended well-woman visits, whose scope is to periodically evaluate women's health and provide preventive care 12.

In this context, we examine the clinical utility of the potential preterm birth risk factors from admitted pregnant women with symptomatic preterm labor. We developed prediction models for preterm birth and described the information obtained from the prediction models to serve as a useful guideline for prolonging pregnancies.

## Materials and Methods

National obstetric specialists and researchers from 20 tertiary hospitals were included in the KOrean Preterm collaboratE Network (KOPEN) registry (Supplementary Figure S1). We recruited pregnant women between 16 and 34 gestational weeks who had symptomatic preterm labor and were admitted into a tertiary care center from March to November 2016. Data collection was completed in September 2017, regardless of whether the admitted pregnant women had given birth. Only data from women who delivered at the participating hospitals were considered.

Pregnant women who had symptomatic preterm labor, cervical incompetence, and premature rupture of membranes were included, and quadruplet multiple pregnancies were excluded. Data were recorded in an electronic case report form (eCRF) using the internet-based clinical research and trial management system at each tertiary hospital.

Collected data included demographics, obstetric and medical histories, and basic laboratory test results, including blood tests and vaginal discharge findings for clinical and basic characteristics. In the questionnaire, sleep quality was evaluated using the Pittsburgh Sleep Quality Index. A pelvic examination was performed to assess the cervical condition, such as presence of bleeding, ripening, opening, and water leakage. A speculum examination was used for fetal fibronectin and vaginal swab culture of microorganisms, if possible. An ultrasound examination was conducted to assess the cervical length and shape, fetal gestational age and weight, presence of anomaly, presentation, amniotic fluid volume, and presence of maternal anatomical abnormalities. Blood serum samples were obtained for assessing the blood count and C-reactive protein (CRP) level.

Preterm labor was defined by uterine contractions lasting 40 to 120 seconds more than two or three times per 20 minutes, or eight times within 60 minutes during electro-fetal monitoring with or without cervical dilatation. Gestational age was assigned based on the last menstrual period and confirmed in the first- and early second-trimester ultrasound examinations. Premature rupture of membranes is defined as the rupture of the fetal membranes before the onset of labor. Cervical incompetence is defined as the inability of the uterine cervix to retain a pregnancy in the second trimester without clinical contractions and/or labor 13.

We categorized three gestational age terms as follows: early preterm birth (before 32 weeks), late preterm birth (32-37 weeks), and term birth (after 37 weeks), based on the WHO preterm birth subgroup categories 5, 6.

### Statistical analysis

R language version 3.3.3 (R Foundation for Statistical Computing, Vienna, Austria), T&F program version 2.6 (YooJin BioSoft, Korea), and IBM SPSS Statistics version 22 (IBM Corp., USA) were used for statistical analyses. Data were expressed as mean ± standard deviation for continuous variables. When variables were normally distributed, the difference between the means of two sample groups, defined by the gestational age at birth, were tested using the Student's t-test or Welch's t-test as appropriate. For non-normally distributed variables, the Mann-Whitney U test was used. For categorical variables, data were expressed as numbers and percentages, n (%). The chi-squared test or Fisher's exact test was performed to test the association between the gestational age subgroups at birth and other categorical variables as appropriate using a contingency table.

### Nomogram

We developed preterm birth prediction models, devised nomograms, and evaluated the discriminatory power of the prediction models using an internal validation procedure.

Receiver operating characteristic (ROC) curve analysis was performed to select potential variables that predict preterm birth defined by gestational age at birth. The discrimination performance of the variables was estimated as the area under the curve (AUC), and *p*-values were computed using the null hypothesis of AUC = 0.5. A *p*-value cutoff of 0.1 was applied to select potential variables that were used in the construction of the prediction model for preterm birth. The cutoff values for the potential variables were selected to maximize the sum of sensitivity and specificity, which were used to transform the variables to binary predictors of preterm birth.

Binary logistic regression analysis was performed to analyze the effect of each potential predictor, selected from basic statistics and ROC curve analysis, of preterm birth. Univariate analysis was performed to investigate the association between outcomes and clinical variables or questionnaire variables. To construct the best-fit prediction model for preterm birth, multivariable logistic regression analysis was performed using a backward variable selection method to determine independent covariates. The criterion for initial input variables was a *p*-value < 0.2 in the univariate analysis. The discriminatory power of the constructed models was estimated using the AUC with leave-one-out cross-validation (LOOCV) performed to estimate the reliability of the constructed model through an internal validation procedure.

To facilitate the practical application of the prediction model in the clinical field, a nomogram was developed. Significant factors from the multivariable logistic regression model were incorporated using a weighted-point system to create a clinical prediction algorithm in a nomogram format. A computer-based application program was developed to facilitate the use of individual probability of preterm birth.

### Decision tree

For practical application of the prediction model in the clinical field, a Classification and Regression Tree (CART) analysis was performed to determine the complex interactions among the candidate predictors in the final tree to build the classification trees.

### Ethics statement

This study was approved by the institutional review board at Ewha Womans University Medical Center (Seoul, South Korea) (IRB No. 2016-04-021), and informed consent was obtained from all participants before enrollment in the study.

## Results

**Figure 1:**
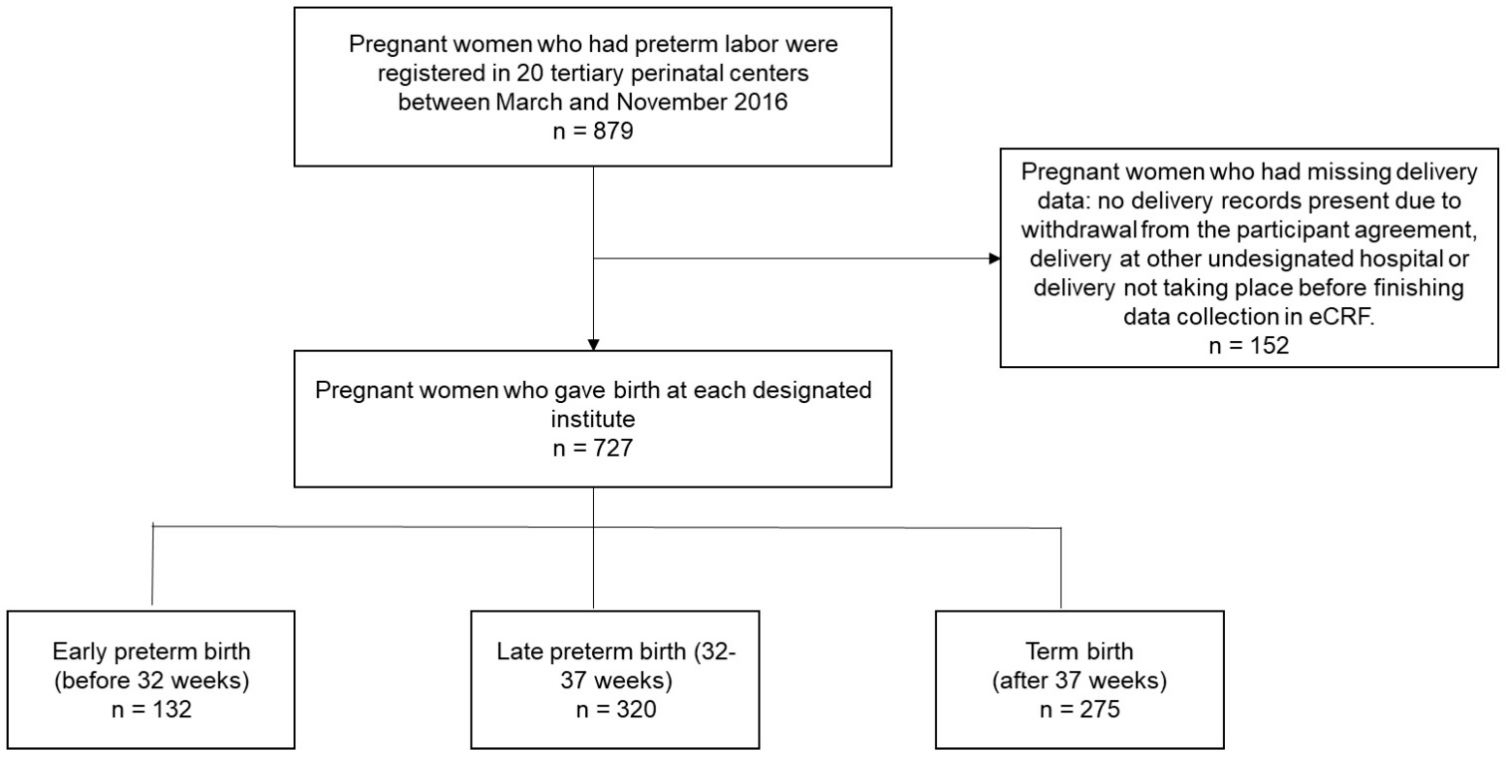
Study design flow chart for all preterm births to identify expected gestational ages of delivery.

In total, 879 pregnant women in preterm labor were registered at the 20 participating tertiary perinatal centers between March 2016 and November 2016 (Figure 1). Of these registered patients, 152 pregnant women had missing birth data such as, no delivery records present due to withdrawal from the participant agreement (22 patients), delivery at another undesignated hospital, or delivery had not yet taken place when data collection was concluded in the eCRF system. Data from the remaining 727 pregnant women who gave birth at a designated institute were analyzed, and the rates of early preterm, late preterm, and term births were 18.16%, 44.02%, and 37.83%, respectively.

The significant factors in maternal characteristics at admission that are associated with preterm delivery are shown in Table 1. With intergroup significance of demographic characteristics, early preterm birth showed higher pre-pregnancy body mass index (BMI), higher rates of pre-pregnancy disease history, earlier gestational age at admission with preterm labor symptoms, lower maternal weight change rates, higher number of stillbirth histories, higher percentage of artificial pregnancies, and higher cerclage histories.

Daily habits shown in Table 2 indicate that term pregnancy is significantly associated with work outside the home; early preterm pregnancy is associated with higher alcohol consumption and poorer sleep quality. There were significant intergroup differences for taking iron supplements and engaging in regular physical activity.

With respect to significant subjective symptoms (pelvic pain, feeling of uterine contraction, sense of pelvic prolapse) and objective signs (vaginal bleeding, water leakage) at admission, early preterm birth was associated with fewer subjective symptoms and more objective signs. The measured biologic characteristics, including shorter cervical length, higher white blood cell count, higher CRP level, and presence of ruptured amniotic membranes, were significantly associated with the early preterm birth group (Table 3).

### Nomogram

**Figure 2:**
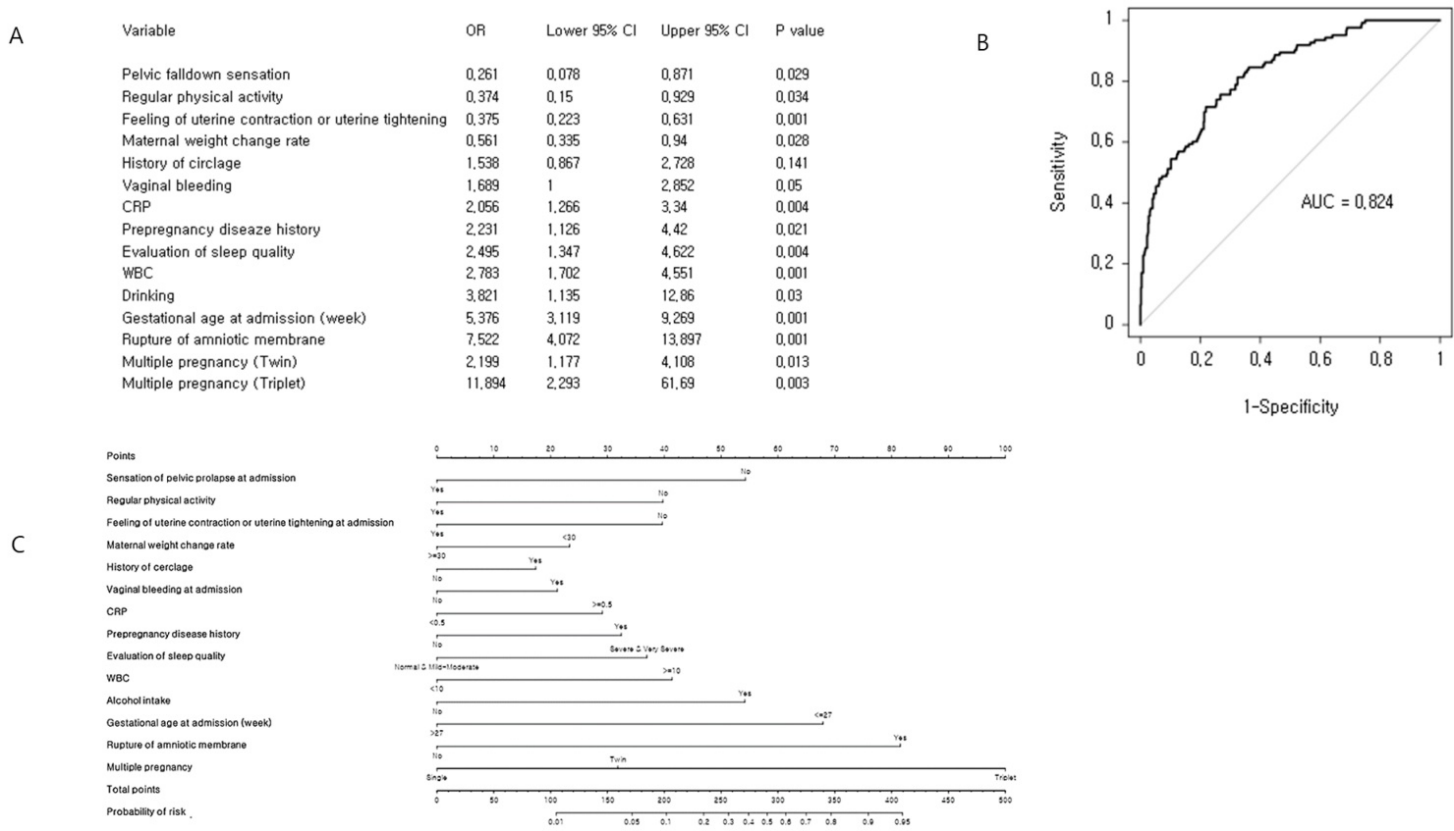
Cross-validation analysis and nomogram for early preterm birth risk: (A) Multiple binary logistic regression analysis for identification of risk factors. (B) Receiver operating characteristic curve of the prediction model. The concordance index for early preterm birth was 0.824 (95% CI: 0.785-0.864). (C) Development of nomogram.

We performed a multivariate logistic regression analysis (Figure 2), which identified 14 significant predictors of preterm birth before completion of 32 weeks of gestation. In order to evaluate the performance of the prediction model internally, we conducted cross-validation using the LOOCV algorithm. The concordance index of the prediction model for preterm birth before completion of 32 gestational weeks was 0.824 (95% CI: 0.785-0.864) and the quantile plot suggests a good estimation of average event rate. Finally, a nomogram was constructed to predict the probability of preterm delivery before completion of 32 weeks of gestation. This model included 14 variables: gestational age at admission, maternal weight change rate, sensation of pelvic prolapse at admission, feeling of uterine contractions or uterine tightening at admission, regular physical activity, history of cerclage, pre-pregnancy disease history, vaginal bleeding at admission, rupture of amniotic membrane, CRP, white blood cell count, alcohol intake, and multiple pregnancy.

**Figure 3:**
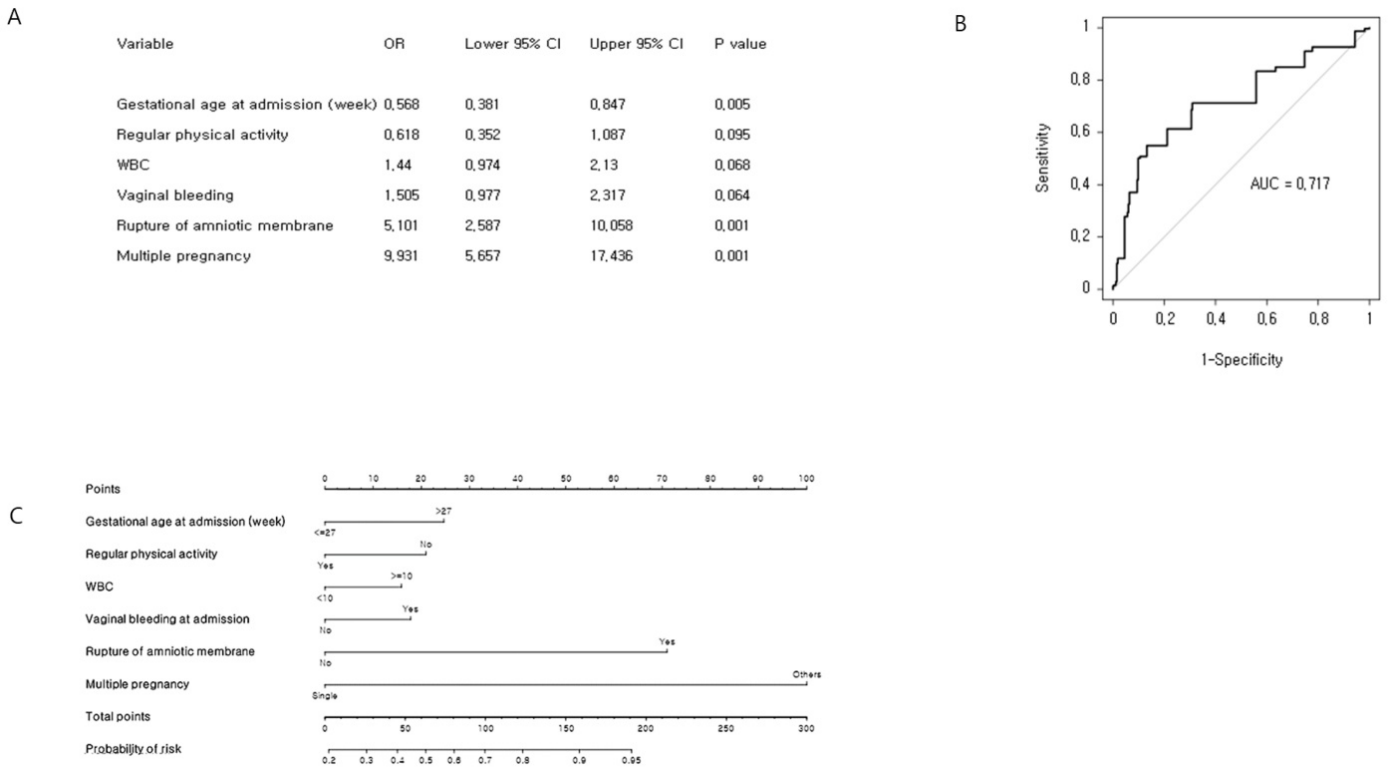
Cross-validation analysis and nomogram of late preterm birth risk factors: (A) Multiple binary logistic regression analysis for identification of risk factors. (B) Receiver operating characteristic curve of the prediction model. The concordance index for late preterm birth was 0.717 (95% CI: 0.675-0.759). (C) Development of nomogram.

Our objective was to predict the estimated time of delivery between 32 and 37 weeks of gestation (Figure 3). A total of 320 (53.8%) preterm babies delivered between 32 and 37 weeks of gestation were identified. The six most significant predictors included gestational age at admission, vaginal bleeding at admission, rupture of membrane, regular physical activity, multiple pregnancy, and WBC, which were determined by univariate logistic regression analysis and multivariate logistic regression analysis. The concordance index of the prediction model for preterm birth between 32 and 37 weeks of gestation was 0.717 (95% CI: 0.675-0.759). We developed an easy-access Microsoft Excel 2013 spreadsheet-based risk predictor (Supplementary Figure S2), where by clicking in the Excel spreadsheet on the cell corresponding to the variable of interest, the probability of individual preterm birth is automatically calculated.

### Decision tree

**Figure 4:**
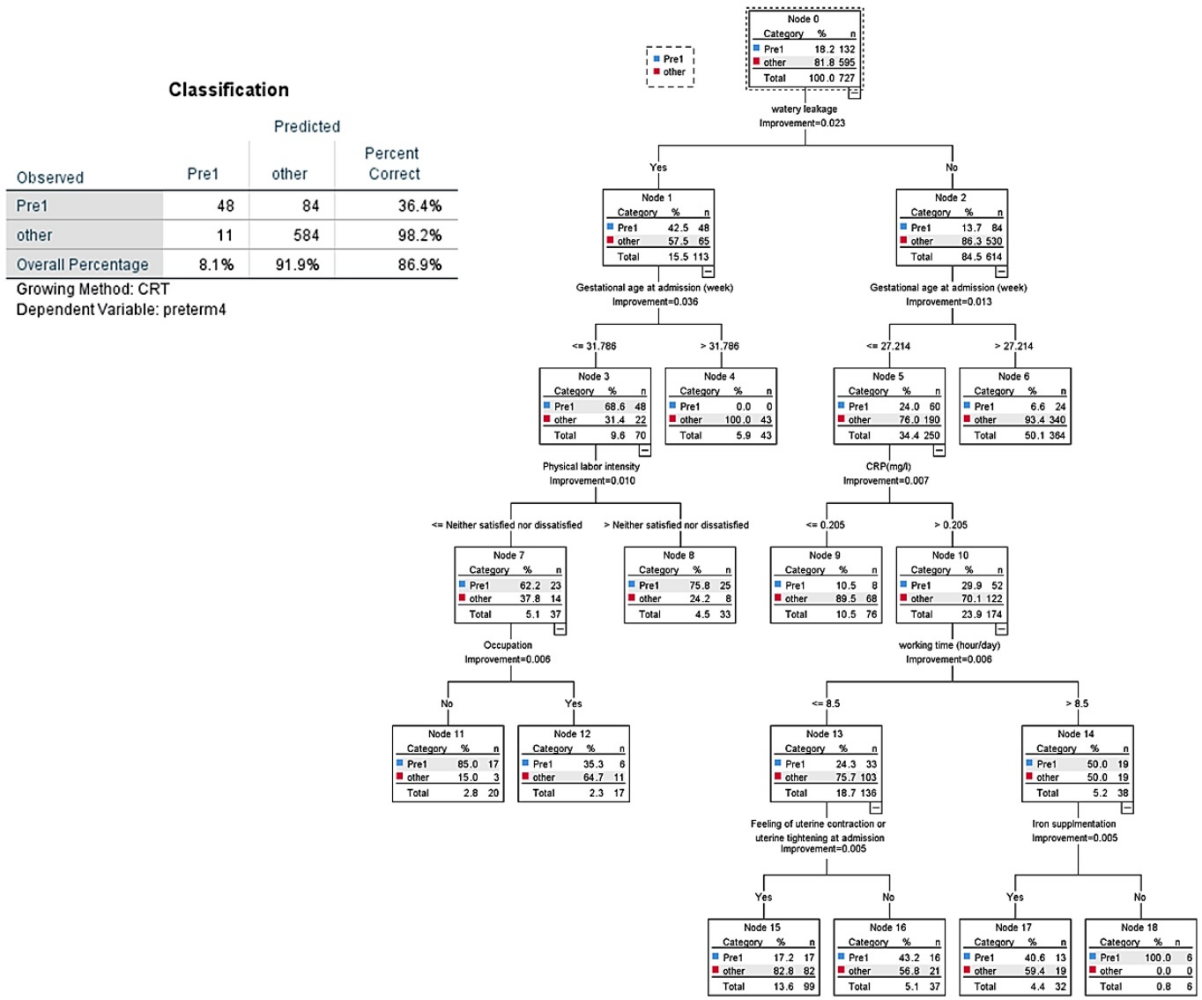
CART decision tree for prediction of early preterm birth at admission (predicted overall percentage 86.9%). Pre1: early preterm birth; other: later preterm birth and term birth.

**Figure 5:**
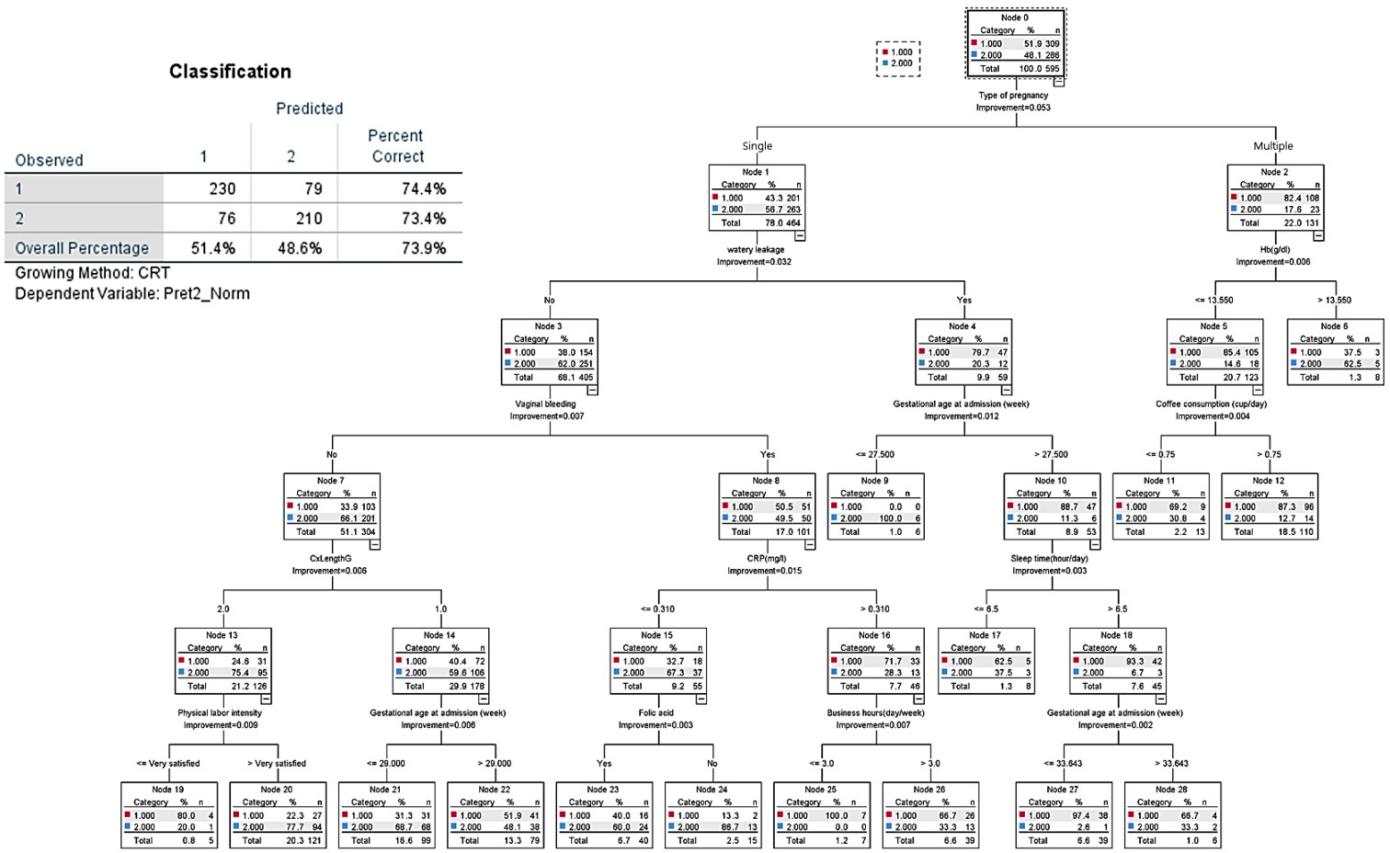
CART decision tree for prediction of late preterm birth at admission (predicted overall percentage 73.9%). 1: Late preterm birth; 2: Term birth.

All variables of tables used in the tested models for the decision tree analysis for the three groups are shown in Figure 4 and 5. In CART analysis, the prediction rate for early preterm birth was 86.9% (Figure 4), with water leakage at admission being the most important node, followed by gestational age at admission. The second node was “no,” then “what if approximately 27 gestational weeks,” then early preterm birth flew down hierarchical nodes like increased CRP level, more than 8.5 hours/day working, less feeling of uterine contractions, and not taking iron supplements.

The decision tree analysis for late preterm birth showed an overall prediction rate of 73.9% (Figure 5). The most important node was “type of pregnancy,” where singleton pregnancy represented 78% of cases, and multiple pregnancy 22%. Of singleton pregnancy, the second and third hierarchical nodes were absence of vaginal bleeding and cervical length larger than 2.5 cm, which tended to prolong term birth**.** In case of multiple pregnancy (23.2% of all pregnancies), 86.4% had preterm birth (late preterm birth, 63.9% versus early preterm birth, 22.5%). CART analysis shows that in the case of multiple pregnancy, the nodes of subjective symptoms such as more labor-like pain and feelings of uterine contractions were associated with late preterm birth; then, nodes of objective signs such as water leakage due to membrane rupture, lower hemoglobin (Hb) levels, and having an occupation at admission were related to late preterm birth.

## Discussion

To our knowledge, this is the first study where predictive models were developed for clinically assessing preterm birth periods (before completion of 32 weeks of gestation, and between 32 and 37 weeks of gestation) using information contained in an eCRF and data obtained at admission, especially subjective symptoms.

Usually, management of patients by obstetricians is based on risk estimation, patient counseling, and decision making. However, commonly used risk estimation methods apply the same risk level to all patients; this approach does not offer the possibility of individualization.

To eliminate this problem and to obtain more accurate predictions, researchers have developed predictive and prognostic tools based on statistical models, which have shown better clinical judgment for predicting probability of outcomes 14.

The first attempt to do this in an obstetric setting had low accuracy and could not be individualized 15. Most predictive models describe risk level for preterm delivery, and some estimate individual probability of preterm birth in cases of suspected preterm birth in the tertiary hospital network setting 16-19. Thus, traditional methods for predicting preterm delivery may be developed based on single factors such as demographic history, obstetric history, or clinical characteristics. Only a few nomograms have been published in obstetrics 17-22; these have primarily focused on suspected preterm delivery and delivery before completion of 32 weeks of gestation at in utero transfer obstetric centers equipped with neonatal intensive care units (NICU) 17-22. The main modification between the previously released models is the integration of cervical length, CRP, and fFN into the novel predictive models 17, 19. In this study, various elements of demographic history, obstetric history, and clinical characteristics were involved in developing our probability model. Previously reported factors such as cervical length, CRP, and fFN were also significantly associated with the preterm birth. For example, the ratio of positive fFN increased to 9.25 (OR=9.25) for the early preterm delivery and the late preterm delivery (OR = 1.50) compared to the term delivery (data not shown). However, the fFN was not included into the predictive model due to too many missing data (about 68%). Cervical length was not selected in the final predictive model during the backward stepwise variable elimination procedure. Interestingly, CRP, which is widely used to monitor inflammatory status and the presence of intrauterine infection 7, 23, was found to be a significant predictor of early preterm birth, but it did not work as a predictor of late preterm birth (Figure 2 and Figure 3).

In the present study, the nomogram-based prediction model may provide information for a personalized assessment of the likelihood of preterm birth by incorporating general risk factors either before completion of 32 weeks of gestation or between 32 and 37 weeks of gestation. We simply developed the nomogram by automatically calculating the probability for individuals using a Microsoft Excel spreadsheet (Supplementary Figure S2). More organization and accurate development of predictive results can be used to visualize the possibility of preterm birth using this predictive model and can evolve into a business that can use mobile applications in a clinical setting for quick decisions.

On the other hand, the proposed decision tree provides a base for developing an antenatal preterm prevention step-by-step guide through the design, implementation, and evaluation of the stages of antenatal lifestyle interventions, such as dietary habits and physical activity levels. In the CART decision tree that we developed, good eating habits, nutrient supplementation and regular physical activity were associated with longer gestational time. Some studies reported that improving diet and physical activity during pregnancy can improve short-term pregnancy outcomes as well as long-term maternal and offspring health 24, 25. During pregnancy, many women are concerned with the health of their infants and are in frequent contact with their healthcare providers. These women may also be more inclined to learn strategies to for healthy lifestyles defined by their eating patterns and physical activity 25-27. Raising awareness and increasing knowledge on the risks associated with lifestyles choices to prevent preterm labor are highly recommended. Maternal education on preterm birth preventive strategies or other health conditions may further contribute toward reducing disease incidence 28. Decision trees make use of useful data-driven software, so there is no empirical cut-point for each variable and no calculations are required; just descend from the beginning to the end of the tree. The most important available outcome variable in the decision tree identifies the most significant relative variable. Thus, this decision tree could provide knowledge of future perspectives on preterm birth.

To this end, nomogram and CART decision trees may be helpful for obstetricians to prepare adequate advice and educate pregnant women. Nomograms are simple and noninvasive visual instruments with a graphical interface that promotes the use of prediction risk models. CART analysis is another type of predictive model with the capacity to account for complex relationships and is relatively easy to use for the clinician. Accurate estimation of preterm birth risk using prediction models improves patient satisfaction after preterm management. In particular, a small change in gestational time by delaying labor could significantly reduce neonatal morbidity and mortality by allowing for an intervention period to accelerate fetal lung maturation 29.

The main strength of our study is providing communication and education as a tool to improve treatment of patients and using currently available preterm birth data and environmental factors involved in a multicenter cohort with prospective recording variables. Our models are based on widely used criteria and a combination of well-known risk factors for preterm birth obtained by using questionnaires on subjective symptoms. Health care providers should evaluate the risks and provide appropriate information for avoiding or managing preterm birth. Our study shows there is a tendency to a prolonged gestational time in patients experiencing subjective symptoms, such as pelvic pain and sense of pelvic prolapse, rather than objective symptoms, such as vaginal bleeding and water leakage.

Another strength of this study is that all the variables in our predictive models are based on data available from the clinical obstetric history, allowing for easy assessment of patients. Preterm births between 32 and 37 weeks of gestation have a relatively lower risk of mortality and morbidity than early preterm births, but the impact on healthcare worldwide may be significant due to their higher risks than full term births 22, 30. The most effective approach to prevent preterm birth is based on individual obstetric history, which makes identifying women at risk for preterm births an important task for clinical care providers.

Many antenatal and postnatal factors modify the risk of preterm birth. Our statistical analyses show there is a selection bias due to fact that the source population consists of a registry of pregnant women with preterm labor symptoms rather than all pregnant women, including those who are asymptomatic 31-33. Another limitation of this study was the lack of data on variables related to the possible mechanism of preterm birth such as fetal fibronectin levels, intraamniotic infections, inflammation related vaginal microbiomes, and cytokine 34, 35, each with consequences associated with gestational age and influenced by execution in each center (e.g. closer monitoring, using antibiotics and steroids).

Despite these limitations, this study developed personalized prediction models of preterm birth risk and an estimation of the delivery period using a wide range of clinical variables obtained at the initial hospital admission. Therefore, these models may assist clinicians and patients in clinical decision making so that appropriate decisions for hospitalization or lifestyle coaching in the outpatient setting can be made. This may also be useful to counsel and educate patients by calculating the overall probability of preterm birth for the individual patient and considering specific risk factors present during critical time points in the gestational period.

## Supplementary Material

Supplementary figures.

## Figures and Tables

**Table 1:** Maternal baseline characteristics (n = 727)

Variable	Subgroup	Gestational age at birth of <32 weeks	Gestational age at birth of 32‒37 weeks	Gestational age at birth of ≥37 weeks	*p*-value
Sample no. (%)		132 (18.16)	320 (44.02)	275 (37.83)	
Age	<30	15 (11.4)	63 (19.7)	49 (17.8)	0.237
	30‒35	73 (55.3)	156 (48.8)	127 (46.2)	
	35‒40	39 (29.5)	91 (28.4)	83 (30.2)	
	≥40	5 (3.8)	10 (3.1)	16 (5.8)	
Pre-pregnancy BMI (kg/m^2^)		21.86±3.47	21.35±3.13	21.14±3.02	0.049^*^
Pre-pregnancy BMI(kg/m^2^)	<18.5	14 (10.7)	45 (14.2)	37 (13.5)	0.849
	18.5‒25.0	98 (74.8)	235 (73.9)	201 (73.4)	
	≥25.0	19 (14.5)	38 (11.9)	36 (13.1)	
Marriage	Married	132 (100)	318 (99.4)	275 (100)	1
Nursing	No	77 (61.6)	216 (69.9)	183 (69.3)	0.215
	Yes	48 (38.4)	93 (30.1)	81 (30.7)	
Medication history	No	108 (81.8)	283 (89)	244 (88.7)	0.084
	Yes	24 (18.2)	35 (11)	31 (11.3)	
Disease history before pregnancy	No	105 (81.4)	285 (89.9)	253 (93)	0.002^**^
	Yes	24 (18.6)	32 (10.1)	19 (7)	
History of preterm birth	No	117 (90)	282 (88.7)	246 (90.1)	0.832
	Yes	13 (10)	36 (11.3)	27 (9.9)	
Gestational age at admission (week)		25.26±4.15	29.18±4.05	27.79±4.44	<0.001^**^
Maternal weight change (kg)		6.1±6.15	8.37±4.9	7.14±4.16	<0.001^**^
Maternal weight change rate (g/week)		24.13±24.2	28.36±16.65	25.42±14.06	0.03^*^
Multiple pregnancy (type of pregnancy)	Single	94 (71.2)	206 (64.4)	258 (93.8)	<0.001^**^
	Twin	34 (25.8)	109 (34.1)	17 (6.2)	
	Triplet	4 (3)	5 (1.6)	0 (0)	
Number of pregnancies		2.02±1.22	1.85±1.15	1.86±1	0.256
Number of deliveries		0.57±0.77	0.4±0.63	0.4±0.57	0.07
Number of live births		0.51±0.74	0.38±0.61	0.37±0.55	0.242
Number of stillbirths		0.05±0.23	0.01±0.14	0.03±0.19	0.019^*^
Number of abortions		0.48±0.97	0.45±0.87	0.46±0.78	0.682
Mode of pregnancy	Natural pregnancy	95 (73.1)	207 (65.1)	258 (94.5)	< 0.001^**^
	*In vitro* fertilization	35 (26.9)	111 (34.9)	15 (5.5)	
History of vaginal bleeding	No	109 (83.8)	273 (85.8)	223 (81.7)	0.389
	Yes	21 (16.2)	45 (14.2)	50 (18.3)	
History of cerclage	No	92 (70.8)	271 (85.2)	224 (82.1)	0.002^**^
	Yes	38 (29.2)	47 (14.8)	49 (17.9)	
History of cervical conization	No	122 (93.8)	306 (96.2)	260 (95.2)	0.54
	Yes	8 (6.2)	12 (3.8)	13 (4.8)	
Uterine anomaly	No	129 (99.2)	315 (99.1)	267 (97.8)	0.444
	Yes	1 (0.8)	3 (0.9)	6 (2.2)	
Delivery mode	Natural delivery	38 (28.8)	112 (35)	139 (50.5)	< 0.001^**^
	Surgical delivery	94 (71.2)	208 (65)	136 (49.5)	
Birth weight (g)		1208.2±565.29	2318.9±425.46	3096.89±421.09	< 0.001^**^
Baby sex	Female	54 (41.2)	142 (44.7)	131 (48.3)	0.379
	Male	77 (58.8)	176 (55.3)	140 (51.7)	

p-value* < 0.05, p-value** < 0.01.

**Table 2:** Maternal characteristics related to daily activities (n = 727)

Variable	Subgroup	Gestational age at birth of <32 weeks (n=132)	Gestational age at birth of 32‒37 weeks (n=320)	Gestational age at birth of ≥37 weeks (n=275)	p-value
Maternal occupation	No	69 (52.3)	157 (49.1)	110 (40)	0.026^*^
	Yes	63 (47.7)	163 (50.9)	165 (60)	
Business hours (day/week)		4.65±1.27	4.58±1.5	4.65±1.23	0.893
Occupation time (hour/day)		8.4±1.49	7.95±1.77	8.18±2.03	0.152
Physical labor intensity	Very satisfied	3 (4.8)	10 (6.2)	8 (5)	0.734
	Somewhat satisfied	10 (15.9)	40 (24.7)	30 (18.9)	
	Neither satisfied nor dissatisfied	16 (25.4)	46 (28.4)	48 (30.2)	
	Somewhat dissatisfied	24 (38.1)	51 (31.5)	54 (34)	
	Very dissatisfied	10 (15.9)	15 (9.3)	19 (11.9)	
Housework strength	Very satisfied	1 (0.8)	5 (1.6)	2 (0.7)	0.666
	Somewhat satisfied	16 (12.1)	40 (12.6)	28 (10.4)	
	Neither satisfied nor dissatisfied	51 (38.6)	126 (39.7)	109 (40.4)	
	Somewhat dissatisfied	44 (33.3)	114 (36)	106 (39.3)	
	Very dissatisfied	20 (15.2)	32 (10.1)	25 (9.3)	
Housework time (hours)		4.22±2.55	4.08±2.48	4.08±2.5	0.889
Housework duration (hour/day)		2.71±2.17	2.86±2.36	2.86±2.26	0.561
Direct smoking	No	112 (84.8)	280 (87.8)	247 (89.8)	0.346
	Yes	20 (15.2)	39 (12.2)	28 (10.2)	
Total smoking amount	No	112 (84.8)	280 (87.8)	247 (89.8)	0.451
	Less than 5 packs	2 (1.5)	9 (2.8)	5 (1.8)	
	More than 5 packs	18 (13.6)	30 (9.4)	23 (8.4)	
Passive smoking	No	99 (75)	250 (78.4)	224 (81.5)	0.31
	Yes	33 (25)	69 (21.6)	51 (18.5)	
Alcohol consumption	No	125 (94.7)	314 (98.4)	266 (96.7)	0.083
	Yes	7 (5.3)	5 (1.6)	9 (3.3)	
Coffee consumption	No	62 (47)	121 (38.2)	104 (38.5)	0.186
	Yes	70 (53)	196 (61.8)	166 (61.5)	
Coffee consumption (cup/day)		1.06±0.37	0.98±0.37	1±0.4	0.136
Eating habits	Meat	19 (14.4)	51 (15.9)	37 (13.5)	0.32
	Vegetables	14 (10.6)	17 (5.3)	19 (6.9)	
	Balanced meal	99 (75)	252 (78.8)	219 (79.6)	
Number of meals (per day)	1-2	44 (33.3)	95 (29.9)	108 (39.4)	0.05
	More than 3	88 (66.7)	223 (70.1)	166 (60.6)	
Food allergy	No	120 (90.9)	300 (94.3)	252 (92.3)	0.378
	Yes	12 (9.1)	18 (5.7)	21 (7.7)	
Time to sleep	Before midnight	107 (81.1)	241 (76.8)	212 (78.8)	0.584
	After midnight	25 (18.9)	73 (23.2)	57 (21.2)	
Sleep time (hours)		7.87±1.63	7.89±1.59	7.81±1.33	0.835
Evaluation of sleep quality	Normal	89 (67.4)	212 (66.7)	194 (71.9)	0.028^*^
	Mild-Moderate	12 (9.1)	56 (17.6)	40 (14.8)	
	Severe & Very Severe	31 (23.5)	50 (15.7)	36 (13.3)	
Nutritional supplement	No	2 (1.5)	5 (1.6)	5 (1.8)	1
	Yes	130 (98.5)	314 (98.4)	270 (98.2)	
Antioxidants	No	116 (89.2)	281 (89.8)	237 (87.8)	0.74
	Yes	14 (10.8)	32 (10.2)	33 (12.2)	
Folic acid	No	29 (22.3)	58 (18.5)	68 (25.2)	0.149
	Yes	101 (77.7)	255 (81.5)	202 (74.8)	
Iron	No	24 (18.5)	31 (9.9)	47 (17.4)	0.011^*^
	Yes	106 (81.5)	283 (90.1)	223 (82.6)	
Multivitamins, minerals	No	78 (60)	180 (57.3)	136 (50.4)	0.115
	Yes	52 (40)	134 (42.7)	134 (49.6)	
Omega 3	No	88 (67.7)	217 (69.6)	196 (72.6)	0.552
	Yes	42 (32.3)	95 (30.4)	74 (27.4)	
Regular physical activity	No	123 (93.2)	290 (91.5)	228 (84.4)	0.006^**^
	Yes	9 (6.8)	27 (8.5)	42 (15.6)	

p-value* < 0.05, p-value** < 0.01.

**Table 3:** Pregnancy characteristics related to symptoms and laboratory test results at admission (n = 727)

Variable	Subgroup	Gestational age at birth of <32 weeks (n=132)	Gestational age at birth of 32‒37 weeks (n=320)	Gestational age at birth of ≥37 weeks (n=275)	p-value
Nausea, vomiting	No	54 (40.9)	121 (38.1)	97 (35.3)	0.528
	Yes	78 (59.1)	197 (61.9)	178 (64.7)	
Pelvic pain	No	36 (27.3)	30 (9.4)	46 (16.7)	<0.001^**^
	Yes	96 (72.7)	290 (90.6)	229 (83.3)	
Feeling of uterine contraction or uterine tightening at admission	No	52 (39.4)	68 (21.2)	61 (22.2)	<0.001^**^
	Yes	80 (60.6)	252 (78.8)	214 (77.8)	
Sensation of pelvic prolapse at admission	No	128 (97)	282 (88.1)	250 (90.9)	0.013^*^
	Yes	4 (3)	38 (11.9)	25 (9.1)	
Low back pain	No	95 (72)	222 (69.4)	195 (70.9)	0.839
	Yes	37 (28)	98 (30.6)	80 (29.1)	
Vaginal discharge	No	71 (53.8)	185 (57.8)	173 (62.9)	0.182
	Yes	61 (46.2)	135 (42.2)	102 (37.1)	
Vaginal bleeding	No	81 (61.4)	236 (73.8)	217 (78.9)	<0.001^**^
	Yes	51 (38.6)	84 (26.2)	58 (21.1)	
Labor-like pain		2.53±2.8	2.7±2.51	2.87±2.57	0.253
Water leakage	No	82 (63.1)	266 (83.6)	261 (95.6)	<0.001^**^
	Yes	48 (36.9)	52 (16.4)	12 (4.4)	
Cervical length (cm)		1.95±1.37	2.01±1.11	2.35±1.14	<0.001^**^
Cervical length (cm)	<2.1	67 (52.8)	164 (52.9)	111 (40.8)	0.008^**^
	2.1‒2.5	10 (7.9)	37 (11.9)	27 (9.9)	
	≥2.5	50 (39.4)	109 (35.2)	134 (49.3)	
fFN	Positive	27(24.1)	50(44.6)	35(31.2)	<0.001^**^
	Negative	5(4.1)	57(46.7)	60(49.2)	
Rupture of amniotic membrane	No	82 (63.1)	266 (83.6)	261 (95.6)	<0.001^**^
	Yes	48 (36.9)	52 (16.4)	12 (4.4)	
Hb level (g/dL)		11.39±1.19	11.51±1.22	11.64±1.14	0.12
WBC count (/mL)		10.86±3.17	9.48±3.91	9.42±2.4	<0.001^**^
WBC count (/mL)	<10	55 (42.3)	202 (63.9)	182 (67.4)	<0.001^**^
	≥10	75 (57.7)	114 (36.1)	88 (32.6)	
CRP level (mg/L)		2.07±8.78	1.42±4.79	1.52±6.8	<0.001^**^
CRP level (mg/L)	<1.5	95 (76)	260 (88.1)	222 (86.4)	<0.001^**^
	1.5-2.0	8 (6.4)	5 (1.7)	6 (2.3)	
	>=2.0	22 (17.6)	30 (10.2)	29 (11.3)	
CRP level (mg/L)	<0.5	55 (44)	193 (65.4)	176 (68.5)	<0.001^**^
	≥0.5	70 (56)	102 (34.6)	81 (31.5)	

Hb, hemoglobin; WBC: white blood cell; CRP, C-reactive protein; fFN, fetal fibronectin. p-value* < 0.05, p-value** < 0.01.

## References

[1] Blencowe H, Cousens S, Oestergaard MZ (2012). National, regional, and worldwide estimates of preterm birth rates in the year 2010 with time trends since 1990 for selected countries: a systematic analysis and implications. Lancet.

[2] Zeitlin J, Szamotulska K, Drewniak N (2013). Preterm birth time trends in Europe: a study of 19 countries. BJOG.

[3] Koullali B, Oudijk MA, Nijman TA, Mol BW, Pajkrt E (2016). Risk assessment and management to prevent preterm birth. Semin Fetal Neonatal Med.

[4] Garcia-Casado J, Ye-Lin Y, Prats-Boluda G, Mas-Cabo J, Alberola-Rubio J, Perales A (2018). Electrohysterography in the diagnosis of preterm birth: a review. Physiol Meas.

[5] World Health Organization. Preterm birth.

[6] World Health Organization. International classification of diseases.

[7] Frey HA, Klebanoff MA (2016). The epidemiology, etiology, and costs of preterm birth. Semin Fetal Neonatal Med.

[8] Gomez R, Romero R, Medina L (2005). Cervicovaginal fibronectin improves the prediction of preterm delivery based on sonographic cervical length in patients with preterm uterine contractions and intact membranes. Am J Obstet Gynecol.

[9] Smith GC (2016). Antenatal betamethasone for women at risk for late preterm delivery. N Engl J Med.

[10] Kawamoto K, Houlihan CA, Balas EA, Lobach DF (2005). Improving clinical practice using clinical decision support systems: a systematic review of trials to identify features critical to success. BMJ.

[11] Bright TJ, Wong A, Dhurjati R (2012). Effect of clinical decision-support systems: a systematic review. Ann Intern Med.

[12] (2018). ACOG Committee Opinion No. 754: The Utility of and Indications for Routine Pelvic Examination. Obstet Gynecol.

[13] (2014). ACOG Practice Bulletin No.142: Cerclage for the management of cervical insufficiency. Obstet Gynecol.

[14] Shariat SF, Karakiewicz PI, Suardi N, Kattan MW (2008). Comparison of nomograms with other methods for predicting outcomes in prostate cancer: a critical analysis of the literature. Clin Cancer Res.

[15] Papiernik E, Kaminski M (1974). Multifactorial study of the risk of prematurity at 32 weeks of gestation. I. A study of the frequency of 30 predictive characteristics. J Perinat Med.

[16] Honest H, Bachmann LM, Sundaram R, Gupta JK, Kleijnen J, Khan KS (2004). The accuracy of risk scores in predicting preterm birth-a systematic review. J Obstet Gynaecol.

[17] Allouche M, Huissoud C, Guyard-Boileau B, Rouzier R, Parant O (2011). Development and validation of nomograms for predicting preterm delivery. Am J Obstet Gynecol.

[18] Schaaf JM, Ravelli AC, Mol BW, Abu-Hanna A (2012). Development of a prognostic model for predicting spontaneous singleton preterm birth. Eur J Obstet Gynecol Reprod Biol.

[19] Mailath-Pokorny M, Polterauer S, Kohl M (2015). Individualized assessment of preterm birth risk using two modified prediction models. Eur J Obstet Gynecol Reprod Biol.

[20] Costantine MM, Fox K, Byers BD (2009). Validation of the prediction model for success of vaginal birth after cesarean delivery. Obstet Gynecol.

[21] Grobman WA, Lai Y, Landon MB (2007). Development of a nomogram for prediction of vaginal birth after cesarean delivery. Obstet Gynecol.

[22] Dukhovny D, Dukhovny S, Pursley DM (2012). The impact of maternal characteristics on the moderately premature infant: an antenatal maternal transport clinical prediction rule. J Perinatol.

[23] Goldenberg RL, Culhane JF, Iams JD, Romero R (2008). Epidemiology and causes of preterm birth. Lancet.

[24] Phelan S (2010). Pregnancy: a "teachable moment" for weight control and obesity prevention. Am J Obstet Gynecol.

[25] Ainscough KM, Lindsay KL, O'Sullivan EJ, Gibney ER, McAuliffe FM (2017). Behaviour change in overweight and obese pregnancy: a decision tree to support the development of antenatal lifestyle interventions. Public Health Nutr.

[26] Steegers-Theunissen RPM (2017). Periconception mHealth platform for prevention of placental-related outcomes and non-communicable diseases. Placenta.

[27] Katz M (2016). Preventing preterm birth. J Perinat Med.

[28] Agger WA, Schauberger CW, Burmester JK, Shukla SK (2016). Developing research priorities for prediction and prevention of preterm birth. Clin Med Res.

[29] Utama DP, Crowther CA (2018). Transplacental versus direct fetal corticosteroid treatment for accelerating fetal lung maturation where there is a risk of preterm birth. Cochrane Database Syst Rev.

[30] Consortium on Safe Labor, Hibbard JU, Wilkins I (2010). Respiratory morbidity in late preterm births. JAMA.

[31] Adams MM, Elam-Evans LD, Wilson HG, Gilbertz DA (2000). Rates of and factors associated with recurrence of preterm delivery. JAMA.

[32] McManemy J, Cooke E, Amon E, Leet T (2007). Recurrence risk for preterm delivery. Am J Obstet Gynecol.

[33] Simonsen SE, Lyon JL, Stanford JB, Porucznik CA, Esplin MS, Varner MW (2013). Risk factors for recurrent preterm birth in multiparous Utah women: a historical cohort study. BJOG.

[34] Fettweis JM, Serrano MG, Brooks JP (2019). The vaginal microbiome and preterm birth. Nat Med.

[35] Lockwood CJ (2002). Predicting premature delivery-no easy task. N Engl J Med.

