# Supplementary material for: The Clinical Usefulness of Predictive Models for Preterm Birth with Potential Benefits: A KOrean Preterm collaboratE Network (KOPEN) Registry-Linked Data-Based Cohort Study
Source: Int J Med Sci. 2020 Jan 1;17(1):1–12. doi: 10.7150/ijms.37626 (PMC6945556; doi:10.7150/ijms.37626)
Supplement: Supplementary file 1 — Supplementary figures. [file ijmsv17p0001s1.pdf]

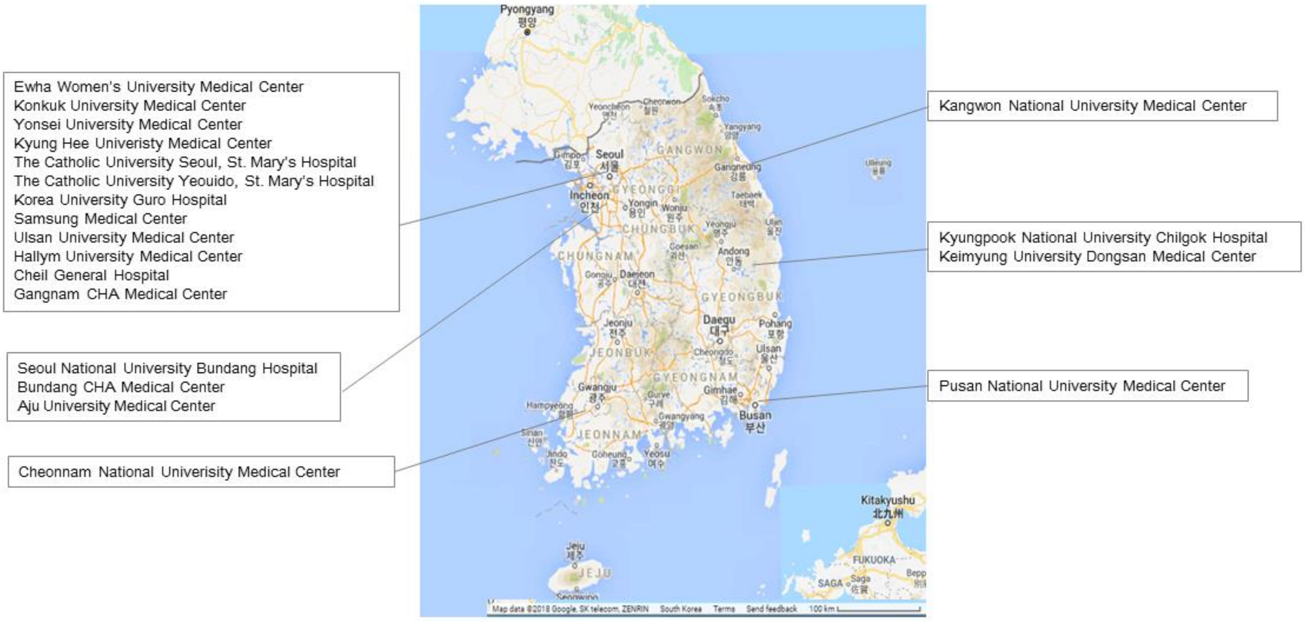

Figure S1. The 20 institutes participating in the nationwide preterm care tertiary hospital KOrean Preterm collaboratE Network (KOPEN) registry.

A

Variables	Select Item (Click)	Points
Pelvic falldown sensation	No	54
Regular physical activity	No	40
Feeling of uterine contraction or uterine tightening	No	40
Maternal weight change rate	<30	23
History of circlage	No	0
Varginal bleeding	No	0
CRP	<0.5	0
Pregnancy disease history	No	0
Evaluation of sleep quality	Normal	0
WBC	<10	0
Drinking	No	0
Gestational age at admission (week)	>27	0
Rupture of amniotic membrane	No	0
Multiple pregnancy	Single	0
Total Points		157
Probability of Risk		0.035

B

Variables	Select Item (Click)	Points
Gestational age at admission (week)	>27	0
Regular physical activity	No	40
WBC	<10	0
Varginal bleeding	No	0
Rupture of amniotic membrane	No	0
Multiple pregnancy	Single	0
Total Points		40
Probability of Risk		0.373

Figure S2. An example of the Excel spreadsheet-based risk predictor for individual probability of preterm birth. (A) First, identify if the mother is undergoing preterm delivery before completion of 32 weeks of gestation; if not, move to (B) estimate the probability of preterm delivery between 32 and 37 weeks of gestation.
